# Length of stay of psychiatric admissions in a general hospital in Ethiopia: a retrospective study

**DOI:** 10.1186/s13033-015-0006-x

**Published:** 2015-03-10

**Authors:** Fikir Addisu, Mekitie Wondafrash, Zeina Chemali, Tariku Dejene, Markos Tesfaye

**Affiliations:** Department of Psychiatry, Jimma University, Jimma, Ethiopia; Department of Population and Family Health, Jimma University, Jimma, Ethiopia; Department of Psychiatry and Neurology, Massachusetts General Hospital, Boston, MA USA; Department of Epidemiology, Jimma University, Jimma, Ethiopia; Center for International Health, Ludwig Maxmillians University, Munich, Germany

**Keywords:** Psychiatric admission, Mental illness, Psychiatric care, Length of hospital stay, Sub-Saharan Africa, Ethiopia

## Abstract

**Background:**

In sub-Saharan Africa, the number of psychiatric beds per population is disproportionately low. Moreover, there is a lack of data regarding the patterns of psychiatric admissions and the factors leading to long psychiatric hospitalization in this region. This study aimed to investigate the average length of stay (LOS) and the factors associated with prolonged hospitalizations.

**Methods:**

A ten-year retrospective chart review of patients admitted to the psychiatric facility of Jimma University Specialized Hospital in southwest Ethiopia was conducted. The medical charts of 846 admissions spanning the period from January 2001 to December 2010 were reviewed. LOS greater than 21 days was considered as a cut-off point for lengthier stay. Bivariate and multivariable logistic regression analyses were conducted to identify factors independently associated with LOS.

**Results:**

The most common discharge diagnoses were schizophrenia and other psychotic disorders (27.6%), and bipolar disorder (23.4%). A global clinical rating taken on discharge showed 90.3% improved outcome. The median (25th, 75th percentiles) LOS was 22 (15, 36) days. Patients with major depressive disorder [aOR = 0.51 (0.32 – 0.81)] and brief psychotic disorder [aOR = 0.52 (0.33 – 0.84)] were less likely than patients with schizophrenia and other psychotic disorders to have long hospital stays. Presence of extrapyramidal side-effects and out of pocket expenditures predicted LOS.

**Conclusions:**

Patients with psychoses and bipolar disorder have lengthier hospital stays burdening the cost of care of psychiatric treatment in a general hospital setting. Our findings call for identifying those cases quickly, attending to their needs with evidence-based efficient treatment and for improving and developing an aftercare system such that the utilization of acute inpatient beds, already a scarce resource, could achieve higher efficiency.

## Background

The state of existing mental health services in low-income countries has been characterized as inadequate, inequitable and inefficient [1]. In particular, the lack of infrastructure in sub-Saharan Africa remains to be a significant barrier to improving mental health services in the region [2]. According to the World Health Organization (WHO), sub-Saharan Africa and Southeast Asia have the lowest number of psychiatric beds per population [3,4]. The majority of psychiatric beds in these regions are situated in large overcrowded mental hospitals [3]. The latter is regarded as inefficient use of the limited mental health resources [1].

Current mental health service models for low-income countries recommend establishment of psychiatric service within primary care and psychiatric inpatient units within general hospitals [5-7]. Ethiopia follows that system of delivery of mental health care. For many years in Ethiopia, mental health services were limited to one hospital situated in the capital, Addis Ababa [8,9]. Recently, a small number of psychiatric inpatient facilities were established in different Ethiopian cities [10].

The first of these regional psychiatric facilities embedded in a general hospital was established in Jimma in 1998 [11]. It opened a psychiatric unit with 6 inpatient beds within Jimma University Specialized Hospital (JUSH). In 2008, the psychiatric facility within the general hospital was enlarged to 26 inpatient beds. It remains the only psychiatric facility for referrals and inpatient care in the southwestern part of Ethiopia serving more than 10 million residents [11]. Peculiar characteristics of this mental healthcare point to inadequate supply of needed medications, the use of older generation psychotropics causing many side effects and a higher level of care subsidized by the government [12,13]. Patients who present to the psychiatric facility with a testimonial letter from their local administration regarding their low socio-economic status are offered subsidized, and often completely free of charge, psychiatric services. Also, with limited specialized manpower, family members are encouraged to stay in the hospital and care for their relatives suffering from mental illness. Of note, rehabilitation facilities and residential programs for people with chronic mental illness do not exist in the region.

Compared to low-income countries, high-income countries decreased their LOS of psychiatric admissions with the deinstitutionalized movement and the development of community based mental healthcare [14-17]. While studies found that shorter psychiatric hospital stays are as therapeutically beneficial as longer ones [16] and save the cost of psychiatric care [18], others have argued that shorter stays are associated with poor outcome including an increased rate of relapse and psychiatric readmissions [14,19]. Inappropriately long hospital stays may result from unavailability or inadequacy of community mental health care [20]. In low-income countries, inadequate or absent community mental healthcare makes short LOS almost impossible to achieve. And although new acute psychiatric inpatient units are being built, this lack of community mental healthcare and rehabilitation services remain major boundaries and form the bottleneck effect to keeping a short LOS [2]. Hence, any policy aiming at reducing LOS should be accompanied by development of community mental health services in all its forms and needed infrastructure.

The average LOS reported by various studies differs among settings and countries. In high-income countries the average LOS in acute psychiatric facilities ranged from 10.5 days to 43 days [21-25] except for Japan, which recorded LOS of 75 days [21]. As to Africa, a report from South Africa indicated that the average LOS at the psychiatric hospitals was higher (219 days) than that of general hospitals (11 days) and district hospitals (7 days) [26]. Two studies from acute psychiatric units of teaching hospitals in Nigeria reported LOS of 23 days and 28.7 days respectively [27,28]. LOS in the state mental hospital in Ethiopia is around 63 days [9].

In addition to all above, various clinical and patient related factors have been found to be associated with long psychiatric hospitalizations. Schizophrenia or non-affective psychosis [9,23,24], the severity of mental illness [29], presence of comorbid physical illness [30,31] and comorbid substance use disorders [29], have been found to predict lengthier LOS. Involuntary admissions and a history of recurrent hospitalizations have also been linked to longer LOS [24,27,28,32].

Socio-demographic factors such as age, gender, marital status, and place of residence were also reported to influence LOS [9,27,28,31,32]. One study found that frequent visits by family members during patients’ psychiatric hospitalizations were associated with an early discharge [31]. This is highly relevant in Ethiopia where families are the only resources for social care and support [8]. Last but not least, subsidized psychiatric care, socio-economic status of patients and their families could negatively impact LOS.

In this context and searching for studies tackling the issue of LOS in Ethiopia, we found only one study describing the pattern of psychiatric admissions to the State mental hospital [9]. There is no data regarding admissions to acute psychiatric wards in general hospital setting. Specifically, there was no identification of factors leading to or associated with prolonged hospital stays in the setting. Such data would provide useful information to future planning of efficient and economically responsible utilization of mental health resources in Ethiopia. A ten year retrospective review of all the admissions to the acute psychiatric ward in JUSH was conducted. The study aimed to describe the patterns of admissions; outcome of patients’ conditions at discharge and factors associated with longer LOS.

## Methods

### Study design and setting

A retrospective review of patient admissions to the psychiatric unit at JUSH between January 2001 and December 2010 was conducted. Patients were referred from the primary care centers or general hospitals. Some patients were self-referred. Some of the admissions may have been involuntary due to the risk to self-harm or harm to others. However, those involuntary admissions are not charted as such due to absent mental health legislation in Ethiopia. Given this, there is no way of finding the exact number of involuntary admissions during the studied period. Pharmacological treatment and psychoeducation were the main interventions provided. No psychotherapy or rehabilitative services were offered during this ten year period chart review.

### Participants and data collection procedure

A total of 968 psychiatric admissions were recorded on the registration book for the period spanning January 2001 to December 2010. Out of 968 admissions reviewed, detailed documentation was available for 846 admissions. The psychiatric nurses under the supervision of a psychiatrist recorded the preliminary data in English. Data was charted on a structured paper format developed for the purpose of this study by the psychiatry nurses and under the supervision of a clinical officer in mental health. The data was then coded and entered into statistical software. Recorded data included, socio-demographic characteristics, place of residence, duration of admission (in days), whether the patient had to pay for the hospital care, diagnosis on discharge, and outcome at discharge (improved, absconded, no change and deceased). The Diagnostic Statistical Manual 4th revision (DSM-IV) diagnoses were categorized into: schizophrenia (including schizophreniform, schizoaffective, delusional disorder and psychotic disorder not otherwise specified), bipolar disorder, major depressive disorder, brief psychotic disorder, and other mental disorders. A senior psychiatry nurse or a psychiatrist assessed outcome at discharge using the Clinical Global Impression (CGI). Comorbidities such as medical – surgical conditions, substance use disorders, and extrapyramidal side-effects were also documented. The use of khat (a commonly abused stimulant substance) was also highlighted. Extrapyramidal side-effects included neuroleptic induced Parkinsonism, dystonia, akathisia and tardive dyskinesias.

### Data analysis

Data was cleaned and analyzed using Statistical Package for Social Science (SPSS) version 16 (SPSS Inc., Chicago, IL, USA). Descriptive statistics such as measures of central tendency, frequencies, and percentages were computed and presented. The median LOS (21 / 22 days) was used as a cut-off for short and long hospital stays. Bivariate logistic regression was done to explore associations between variables of interest and LOS. Predictors of LOS were assessed using the multivariate logistic regression model to adjust for potential confounding effects with list-wise deletion for missing values. Three patients who died during admission were excluded from the logistic regression analyses. Longer hospital stay was included in the logistic model as the dependent variable. Statistical significance was declared at *p*-value of less than 0.05.

### Ethics statement

The Ethical Review Board of College of Public Health and Medical Sciences, Jimma University granted ethical approval. The data was collected with no personal identifiers at all stages of the study.

## Results

### Background characteristics

The majority of patients were male (58.2%) and Muslim (55.3%). The patients’ age ranged from 7 to 85 years with mean (±SD) of 27 (±10.4) years. Nearly half of the patients were aged between 20 and 29 years. The majority of patients were either single (54.9%) or married (41.2%). Farming was the most common occupation (26.4%). Twenty-nine percent had some primary education. The majority of patients (58.3%) lived outside of Jimma city. The cost of hospital care was covered by the government for over 75% of the patients (Table 1).

**Table 1:** **Background characteristics of psychiatric admissions to Jimma University Specialized Hospital**

**Characteristics**	**Number**	**%**
Sex (n = 846)		
Male	492	58.2
Female	354	41.8
Age in years (n = 846)		
Less than 20	164	19.4
20 – 29	415	49.1
30 – 39	168	19.9
40 and above	99	11.7
Marital status (n = 461)		
Single	253	54.9
Married	190	41.2
Divorced	12	2.6
Widowed	6	1.3
Occupation (n = 390)		
Civil servant	55	14.1
Farmer	103	26.4
Housewife	75	19.2
Student	98	25.1
Unemployed	42	10.8
Trader	17	4.4
Education (n = 317)		
Illiterate	63	19.9
Primary	92	29.0
Secondary	70	22.1
Tertiary	92	29.0
Religion (n = 447)		
Orthodox Christian	144	32.2
Muslim	247	55.3
Protestant	52	11.6
Other	4	0.9
Place of residence (n = 842)		
Within 5 km of Jimma city	351	41.7
≥5 km outside of Jimma city	536	58.3
Patient paid for hospital services (n = 832)		
No	646	77.6
Paid half of the cost	54	6.5
Paid all of the cost	132	15.9

### Patterns of admissions

**Figure 1:**
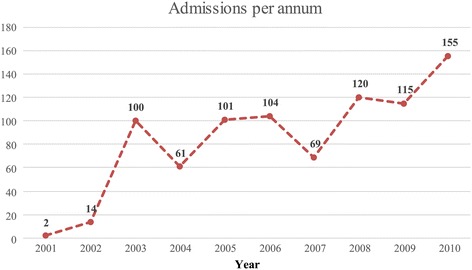
**Number of psychiatric admissions per year 2001 – 2010 at Jimma University Specialized Hospital, Jimma.**

The annual number of psychiatric admissions increased from two in 2001 to 155 in 2010. The sharp increase in the annual admissions between 2002–2003 (from 14 to 100) paralleled the increase in bed capacity to 12 beds on the inpatient unit. Similarly, the increase in annual admission from 69 to 120 between the years 2007 and 2008 overlaps with the expansion of the unit beds to 26 (Figure 1).

### Clinical characteristics and outcome

Data on discharge diagnoses was available only for 838 of the patients. The most common diagnoses on discharge were schizophrenia and other psychotic disorders (27.6%), and bipolar disorders (23.4%). Psychiatric comorbidities were reported in 11.3% charts. Medical-surgical comorbidities complicated 12.6% patients. Reported extrapyramidal side-effects due to medications occurred in 18.7% patients. Khat (33.8%) and alcohol (14.5%) were the most common substances used. Based on a global clinical rating of outcome on discharge 90.3% showed improved outcome while 1.4% had no change. The three deaths on the psychiatry ward were due to medical illness (Table 2).

**Table 2:** **Clinical characteristics and outcome of psychiatric admissions to Jimma University Specialized Hospital**

**Characteristic**	**Number**	**%**
Discharge diagnosis (n = 838)		
Schizophrenia‡	231	27.6
Bipolar disorder	196	23.4
Major depressive disorder	181	21.6
Brief psychotic disorder	153	18.3
Other mental disorders§	77	9.2
Co-morbid psychiatric disorders (n = 699)		
Yes	79	11.3
No	620	88.7
Comorbid medical illness (n = 731)		
Yes	92	12.6
No	639	87.4
Khat use (n = 631)		
Yes	213	33.8
No	418	66.2
Alcohol use (n = 605)		
Yes	88	14.5
No	517	85.5
Extrapyramidal side-effects (n = 732)		
Yes	137	18.7
No	595	81.3
Outcome (n = 846)		
Improved	764	90.3
Absconded	20	2.4
Deceased	3	0.4
No change	12	1.4
Self-discharge	16	1.9
Referred	31	3.7

‡Including schizophreniform, schizoaffective, delusional disorder, psychotic disorder not otherwise specified.

§Anxiety disorders, somatoform disorders, adjustment disorder, delirium, dementia, epilepsy, developmental disorders.

### Length of hospital stays (LOS)

The LOS had a positively skewed distribution and ranged between one day and 261 days. The median (25th, 75th percentiles) length of hospital stay was 22 (15, 36) days. Only 1.2% (10 / 845) admissions were longer than three months and three admissions were for over 6 months duration. The mean LOS for each diagnostic category was 34.2, 32.6, 24.5, 23.7, and 25.1 days for schizophrenia and other psychotic disorders, bipolar disorder, major depressive disorder, brief psychotic disorder, and other mental disorders respectively.

### Factors associated with LOS

Discharge diagnosis, extrapyramidal side–effects and out of pocket expenditures for the hospital care were associated with LOS (Table 3). On multivariable logistic regression patients with major depressive disorder [aOR = 0.51 95% CI (0.32 – 0.81)] and brief psychotic disorder [aOR = 0.52, 95% CI (0.33 – 0.84)] had shorter hospital stays compared to those with a diagnosis of schizophrenia and other psychotic disorders. In contrast, patients with bipolar disorder did not have significant differences in LOS compared to those with schizophrenia and other psychotic disorders [aOR = 0.97, 95% CI (0.61 – 1.52)]. The presence of medication induced extrapyramidal side-effect was associated with longer hospital stay [aOR = 1.53, 95% CI (1.03 – 2.27)]. Similarly patients who paid for the cost of the hospital care either partially [aOR = 0.47; 95% CI (0.25, 0.88)] or wholly [aOR = 0.57; 95% CI (0.36, 0.89)] were likely to have shorter LOS compared to patients who got the hospital care free of charge (Table 4).

**Table 3:** **Bivariate logistic regression for factors associated with long hospital stay (≥22 days) among psychiatric admissions to Jimma University Specialized Hospital**

**Characteristic**	**Long hospital stays**		**Unadjusted odds ratios (95% CI)**	**P-value**
	**Number**	**%**		
Sex (n = 845)				0.972
Male	271	55.1	1	
Female	194	55.0	1.00 (0.76, 1.31)	
Age in years (n = 845)				0.552
<20	83	50.9	1	
20 – 29	228	54.9	1.18 (0.82, 1.69)	
30 - 39	95	56.5	1.25 (0.81, 1.93)	
≥40	59	59.6	1.42 (0.86, 2.36)	
Marital status (n = 460)				0.812
Single	133	52.8	1	
Married	99	52.1	0.97 (0.67, 1.42)	
Divorced	8	66.7	1.79 (0.53, 6.09)	
Widowed	3	50.0	0.90 (0.18, 4.52)	
Place of residence (n = 843)				0.706
Within 5 km of Jimma	159	51.8	1	
≥5 km outside of Jimma city	304	56.7	1.06 (0.80, 1.39)	
Discharge diagnosis (n = 838)				<0.001
Schizophrenia‡	148	64.1	1	
Bipolar disorder	123	62.8	0.95 (0.64, 1.40)	
Major Depressive disorder	84	46.4	0.49 (0.33, 0.72)	
Brief psychotic disorder	73	48.0	0.51 (0.34, 0.78)	
Other mental disorders§	36	46.8	0.49 (0.29, 0.83)	
Comorbid medical illness (n = 698)				0.267
No	326	52.6	1	
Yes	45	57.7	1.29 (0.83, 2.00)	
Comorbid psychiatric disorder (n = 730)				0.394
No	335	52.5	1	
Yes	54	58.7	1.23 (0.76, 1.98)	
Khat use (n = 630)				0.163
No	214	51.2	1	
Yes	121	57.1	1.27 (0.91, 1.77)	
Alcohol use (n = 604)				0.329
No	270	52.3	1	
Yes	51	58.0	1.26 (0.80, 1.98)	
Extrapyramidal side-effects (n = 731)				0.015
No	304	51.2	1	
Yes	86	62.8	1.61 (1.10, 2.36)	
Patient paid for hospital services (n = 831)				0.002
No	372	57.7	1	
Paid half of the cost	21	38.9	0.47 (0.26, 0.83)	
Paid all of the cost	59	44.7	0.59 (0.41, 0.87)	

‡Including schizophreniform, schizoaffective, delusional disorder, psychotic disorder not otherwise specified.

§Anxiety disorders, somatoform disorders, adjustment disorder, delirium, dementia, epilepsy, developmental disorders.

**Table 4:** **Multivariable logistic regression for factors associated with long hospital stay (≥22 days) among psychiatric admissions to Jimma University Specialized Hospital**

**Variable**	**β (S.E)**	**Wald**	Odds ratio (95% CI)*	**P-value**
Discharge diagnosis		22.67		<0.001
Schizophrenia‡			1	
Bipolar disorder	−0.03 (0.23)		0.97 (0.61 – 1.52)	
Major Depressive disorder	−0.67 (0.23)		0.51 (0.32 – 0.81)	
Brief psychotic disorder	−0.65 (0.24)		0.52 (0.33 – 0.84)	
Other mental disorders§	−1.12 (0.34)		0.33 (0.17 – 0.64)	
Extrapyramidal side-effects		4.41		0.036
No			1	
Yes	0.42 (0.20)		1.53 (1.03 – 2.27)	
Patient paid for hospital services		10.67		0.005
No			1	
Paid half of the cost	−0.75 (0.32)		0.47 (0.25 – 0.88)	
Paid all of the cost	−0.57 (0.23)		0.57 (0.36 – 0.89)	

*The constant for the model = 0.593.

‡Including schizophreniform, schizoaffective, delusional disorder, psychotic disorder not otherwise specified.

§Anxiety disorders, somatoform disorders, adjustment disorder, delirium, dementia, epilepsy, developmental disorders.

## Discussion

The annual number of admissions had steadily increased over the ten-year period. Psychotic disorders and severe mood disorders accounted for the majority of admissions. The median duration of hospitalization was 22 days. Discharge diagnosis, the presence of medication induced extrapyramidal side-effects, and financial coverage of hospitalization by the government were predictors of longer hospital stays.

The increase in the yearly number of psychiatric admissions for the ten-year period may have been related to an increase in public awareness regarding the availability of inpatient psychiatric services at JUSH. In addition, the striking increase in admission rates overlapping the increase in bed capacity could be an indirect evidence for the existing unmet mental health service needs in the region. Studies from rural Ethiopia have previously reported that less than 10% of persons with severe mental illness had contact with the mental health system [33].

The most common diagnoses encountered in this study is consistent with the reports by previous studies from the state mental hospital in Addis Ababa [9] as well as from most other settings where schizophrenia and/or non-affective psychoses were the most common diagnosis [21,25,27,28,34]. The predominant diagnoses of bipolar disorder and major depressive disorder is consistent with the pattern of outpatient diagnoses published earlier in the same setting [12]. The lack of consistent supply of psychoactive medications with proven efficacy in mood disorders such as lithium carbonate, and antidepressant medications during the ten-year period could have resulted in increased rates of relapse and hospitalization among the patients with mood disorders [12,13]. Indeed, this result needs further elucidation and future research would provide crucial information on the consequences of suboptimal drug supply at the national and regional level on the mental health system.

The median LOS in JUSH though comparable to findings from similar settings in sub-Saharan Africa [27,28] is higher than LOS reported from the developed countries [26]. The median LOS of 63 days reported from Amanuel hospital is consistent with reports from other settings that psychiatric admissions to general hospitals being shorter than admissions to psychiatric hospitals [15,26]. Moreover, the majority (56%) of admissions to the mental hospital were accounted for by schizophrenia compared to the 27.6% in our study.

Several studies have found that a diagnosis of schizophrenia or non-affective psychoses to be predictors of long hospital stays [9,23,24,28]. The chronic nature of psychotic disorders compared to mood disorders might explain this finding although at times it is hard to make a clear differentiation between the two. In our study, a lengthy LOS was also reported for bipolar disorders, a unique finding when compared to other studies, which reported for example a median LOS of 18.0 days for bipolar patients on quetiapine treatment [35]. In that study, the patients received medications proven to be very effective in the treatment of bipolar disorder leading to shorter LOS. In JUSH as it is the case in other rural regions of Ethiopia, managing bipolar disorder without the consistent use of medications with proven efficacy is a major challenge clinicians face on a daily basis. Our findings could be the reflection of this lack of consistent use of efficacious medications in patients with bipolar disorders and hence the unexpected increase in LOS in JUSH.

The lack of association between long hospital stays and medical comorbidities in this study contrasts with reports from other settings [30,36]. The incomplete medical records or perhaps under-diagnosis of medical conditions might explain this lack of association. On the other hand, our study agrees with reports that the occurrence of adverse side effects was linked with increased LOS among medical and surgical patients [37].

These side effects needed additional treatment and observation by hospital staff. The occurrence of extrapyramidal side-effects could be directly linked to the fact that in low-income countries, and especially rural settings, classical neuroleptics, such as haloperidol, are used to treat psychiatric conditions. These older generation neuroleptics carry a higher side effects profile compared to the new generation atypical neuroleptics, though more expensive.

The cost of atypical antipsychotics use lessening extrapyramidal side-effects in settings where psychiatric beds are scarce should be compared to the cost of an extended stay. This head-to-head comparison would then dictate future policies and treatment plans and perhaps vacating hospital beds earlier. To the best of the authors’ knowledge such study was not yet conducted or published in Ethiopia or the African continent.

Other researchers have reported that public funding to be associated with longer hospital stays [35]. The longer hospital stays for admissions paid by the government is probably linked to the socio-economic status of the patients. As patients who are entitled to government paid hospital services must have produced testimonial letter that they are very poor, they often tend to be unemployed, lacking financial support from their relatives, or even are homeless. Discharging them could be a sophisticated if not impossible task. The lack of psychosocial aftercare including residential facilities, supported employment and rehabilitative services in the region hinders an earlier discharge from acute psychiatric facilities [2]. Consequently, the already small number of psychiatric beds available shrink gradually as patients with chronic mental illness fail to be discharged and continue to occupy the hospital units for several months or even years.

The lack of association between LOS and marital status has also been reported from Amanuel hospital [9]. Despite studies from other settings showing that married patients had shorter hospital stays than unmarried ones [27,31], having relatives stay with patients, regardless of their marital status, on the units in JUSH might have favored a speedy recovery and hence shorter LOS. Other researchers had results to support this stating that more frequent family visits were associated with short hospital stays [31]. Family support and the human connectedness factor provide a huge strength to the patient and the treatment team and should be looked at as a precious, readily available, resource to capitalize on in settings such as Jimma or other rural and developing countries.

### Study limitations

First, this study is based on a retrospective review of the database in JUSH. Our lack of control on the transcriptions in the charts has limited the information we can access to shed a better light on the topic of LOS. Secondly, we encountered incomplete charts and had a large number on missing data on background demographics such as occupation, marital status and education. This lack of information could have altered our results and skewed our results. Thirdly, our results could not be generalizable to other settings. This study involving one setting in rural Ethiopia could not set the example for the whole country or other countries in Africa. Finally, causal relationships could not be drawn from our data analysis and our study did not answer whether shorter stays are associated with higher relapse and frequent re-hospitalization.

## Conclusions

Despite the study limitations enumerated above, this study is first of its kind to explore the topic of LOS in a general hospital setting in Ethiopia such as JUSH. The findings provide useful information on mental health services in that region with data collected over a decade. There is no doubt our results could weigh on the direction of future studies crucially needed in this area. The patterns of common diagnoses for hospitalization as well as factors predicting longer LOS should inform policy makers and hospital managers on cost-effective programs and their impact on psychiatric healthcare delivery. Indeed, the observed predictors of longer LOS could be modifiable such as improving a constant supply of psychotropic and their availability to patients in addition to developing a wide range of psychosocial interventions to parallel at a lower cost the expansion of acute psychiatric services in the region. Our findings call for the start of a debate to improve the current system of care and to efficiently utilize the limited number of psychiatric beds. In particular there is a pressing need to improve the availability of evidence based treatment options for schizophrenia and mood disorders and most importantly to establish sound psychosocial services for the poor patients with mental illness in Ethiopia.
